# Gender Differences in Lifestyle and Mental Health among Senior High School Students in South Korea

**DOI:** 10.3390/ijerph182010746

**Published:** 2021-10-13

**Authors:** Hyunlye Kim, Kwang-Hi Park, Suin Park

**Affiliations:** 1 Department of Nursing, College of Medicine, Chosun University, Gwangju 61452, Korea; hlkim5207@chosun.ac.kr; 2 College of Nursing, Gachon University, Incheon 21936, Korea; parkkh@gachon.ac.kr; 3 College of Nursing, Kosin University, Busan 49267, Korea

**Keywords:** gender differences, adolescence, lifestyle, mental health

## Abstract

Gender differences in health outcomes have long been a concern worldwide. We investigated the gender differences in the lifestyle and mental health status of senior students in general high schools who were preparing for college entrance exams. This secondary analysis was based on data from the 14th Korea Youth Risk Behavior Web-Based Survey (2018). The data of 8476 students in the third year (12th grade) of general high school, among a total of 60,040 middle and high school students nationwide, were analyzed. Mean and standard error (SE) and weighted percentage data were obtained, and the Rao–Scott χ^2^ test was performed. Boys reported more risky behaviors related to drinking and smoking, while girls had more negative perceptions of their bodies and overall health. In addition, girls showed unhealthier lifestyle-related behaviors (breakfast, physical activity, weight control) and greater vulnerability to poor mental health, including lower sleep satisfaction, stress, depression, and suicidal thoughts. Our results suggest that education and health institutions should consider the needs of each gender separately. A gender-specific approach to maintaining healthy lifestyles and good health status among senior high school students is highly recommended.

## 1. Introduction

Gender, as a continuum of socioculturally constructed roles and behaviors, is one of the most important determinants of health outcomes [1]. Gender differences in health outcomes have long been of concern. In general, women live longer than men but suffer from poorer health [2]. Men have higher rates of life-threatening chronic diseases at younger ages, as well as higher rates of externalizing mental health problems and substance use disorders, while women have higher rates of chronic debilitating conditions and internalization of mental problems [3]. Gender differences in health outcomes can be attributed to structural (age, socioeconomic status, family composition, etc.), behavioral (diet, exercise, smoking, alcohol consumption, etc.), and psychosocial (stress, life events, resources, etc.) factors [4]. These differences depend not only on the type of health indicator used but also on the life stage analyzed, and even the country in which the study is being conducted [3]. This study explored gender differences in lifestyle and mental health by analyzing the data of a nationally representative sample of South Korean adolescents.

Lifestyle behaviors are everyday activities related to an individual’s values, knowledge, and norms, shaped by the wider socioeconomic and cultural context; they affect overall health [5]. Diet, activity, smoking, and drinking alcohol are associated with obesity and chronic disease, which are strongly influenced by attitudes and behaviors that differ between the sexes [6]. In a sample of adolescents from a study based on US Project EAT I-IV (Eating and Activity in Teens and Young Adults) data, the prevalence of body dissatisfaction, low vigorous physical activity, and unhealthy weight control behavior was higher in girls, and there was no gender difference in obesity and fast food intake [7]. According to a large-scale face-to-face interview survey of adolescents from three Asian cities (Hanoi, Shanghai, and Taipei), the prevalence of smoking-only, drinking-only, or both was significantly higher in boys [8]. In a study using a large sample of Chinese adolescents, the high rate of suicidal ideation was associated with health risk behaviors such as smoking, binge drinking, and fighting among high school students in Beijing, and differences according to grade and gender were identified [9]. In a study of 1000 students from seven high schools in Turkey, a negative correlation was observed between problematic Internet use and a healthy lifestyle; gender also played a role [10]. As such, the pattern of gender differences according to the type of lifestyle is diverse, and lifestyle is relevant to mental health. Therefore, exploring the lifestyles of adolescents is important in the public health field.

Mental health problems addressed to adolescents include self-awareness, sleep problems, stress, depression, and suicidal ideation. In a study examining gender differences in body and self-perceptions of Swedish high school students, girls were less satisfied with themselves and their appearance and felt inferior to their male partners [11]. Among Turkish senior high school students, exam anxiety was found to have a negative effect on sleep quality [12]. A group of Spanish adolescents found that the mental health of girls was more affected by COVID-19 confinement than boys, exhibiting more anxiety, less self-esteem, more problems with emotional regulation, and more physical dissatisfaction [13]. Depression, anxiety, and suicidal ideation were also reported more frequently among girls in studies of a sample of high school students from China and Jordan [9,14]. In a nationwide sample of adolescents in New Zealand, more girls reported poor sleep hygiene, and consequently a poor sleep quality [15]. Thus, it seems that girls generally have poorer mental health than boys, but the opposite may be true in certain circumstances. In a sample of Turkish senior high school students, no gender differences were found in the level of exam anxiety and related factors [12]. To identify gender differences in adolescent health, it is necessary to perform detailed examinations considering the specific setting.

This study tried to explore the gender differences in health behaviors and outcomes by focusing on the characteristics of the Korean adolescent group. The college enrollment rate of high school students reached 72.5% in 2020 in Korea [16], and the rate of upper secondary and tertiary education is higher in Korea than in all other OECD countries [17]. Academic achievement is highly valued in Korean society, which has contributed to a high-pressure environment for students [17]. Academic pressure has been shown to be the main risk factor for suicide among Korean adolescents [18]. In a hypercompetitive academic atmosphere such as Korea, the academic stress of senior high school students preparing for college entrance exams is at a peak [19], which leads to negative mental health outcomes [9,20]. A study of senior high school students in Taiwan also revealed relatively high rates of fatigue, sleep problems, daytime sleepiness, and depression, in association with academic stress or pressure [21]. A study including a representative sample of young Japanese people indicated a correlation of unhealthy lifestyle with poor mental health; characteristics associated with poor mental health included female gender and being a senior high school student [22]. According to a study of senior high school students in the Philippines, nomophobia and smartphone addiction predicted lifestyle in adolescents, and girls were more prone to nomophobia than boys [23]. Therefore, there is a need for more attention to and care for senior high school students, whose lifestyle and mental health can be greatly threatened by high academic stress.

Adolescence is a decisive developmental stage, during which individuals experience significant physical, psychological, emotional, and social changes [24]. A healthy lifestyle and good mental health during this period are key for establishing positive health behavior as an adult [25,26]. Data from a longitudinal survey followed from 1998 to 2016 during the adolescent-to-adult transition show that an unhealthy lifestyle formed in the early developmental period consistently continues and worsens into adulthood [7]. In particular, late adolescence, as the period just before adulthood, has a decisive influence on subsequent health behaviors. Therefore, a healthy lifestyle and maintenance of daily health is important, even during college entrance exams. We analyzed nationally representative data on the health behaviors of about 15% of all adolescent (middle and high school) students in South Korea, by gender and grade. This study aimed to investigate gender differences in the lifestyles and mental health status of senior students in general high schools preparing for college entrance exams.

## 2. Materials and Methods

### 2.1. Study Design and Data Source

We conducted a secondary analysis of national South Korean data. The data source was the 14th Korea Youth Risk Behavior Web-Based Survey (KYRBS), published by the Korea Centers for Disease Control and Prevention (currently Korea Disease Control and Prevention Agency, KDCA) [27]. The KYRBS is an ongoing web-based survey to monitor health behaviors among middle and high school students. This survey was conducted from September to November 2018 using anonymous self-administered questionnaires. We handled and analyzed this data source in accordance with the KDCA regulations on the disclosure and management of raw data.

### 2.2. Study Sample

The target population for the KYRBS was middle and high school students in South Korea. The 2018 KYRBS used a complex sampling design based on weighting, stratification, and clustering to derive representative results. The sample was drawn from 400 middle schools and 400 high schools, refined to 5 of each for the 17 cities and provinces. Schools and classes were randomly selected according to the stratified sampling method. All students in each selected class were surveyed. Long-term absentees, special needs children, and students with literacy disabilities were excluded from the sample. In total, 62,823 middle and high school students from 800 schools were targeted, of whom 60,040 (95.6%) actually participated in the survey; 8476 (4131 boys and 4345 girls) of these students, in the third year (12th grade) of general high school, were analyzed. Of the 8476 students in the 12th grade, 51.0% (*n* = 4131) were boys and 49.0% (*n* = 4345) were girls. The mean ± SE age was 17.45 ± 0.01 years (range: 12–18 years), and most students were aged either 17 or 18 years. Most of the students (92.5%) lived with their family. Self-reported economic status and academic achievement were usually medium (47.7% and 31.9%, respectively). Less than half of the students reported that they had received education on nutrition and eating habits (30.4%) and alcohol (27.7%), whereas more than half had received education on smoking (58.7%) in the last year. Body mass index (BMI) was normal in 54.2% of the total population. A total of 35.3% of the respondents perceived themselves as fat, and 41.6% of the respondents perceived themselves as healthy.

### 2.3. Variables

#### 2.3.1. General Characteristics

General characteristics of interest included gender, age, living situation, economic status, subjective academic achievement, health education at school, BMI, perceived body image, and perceived health status. The original response options of living situation were “living with family”, “living with relatives”, “living alone or with friends”, “dormitory”, and “orphanage”. These five options were recategorized to “living with family” and “not living with family” for analysis since the proportion of “living with family” was 92.5% and the proportion of the other four categories, even combined, was very small. The response options for household economic status and subjective academic achievement in the last 12 months were “high”, “medium-high”, “medium”, “medium-low”, and “low”. With regard to health education at school, participants were asked whether they had received education about nutrition/eating habits, drinking, and smoking in the last 12 months. BMI was calculated from self-reported weight and height and was divided into categories according to the definitions proposed by the World Health Organization Regional Office for the Western Pacific: underweight (<18.5), normal weight (≥18.5, <23.0), and overweight (at risk ≥23.0, <25.0; obese I ≥ 25.0, <30.0; obese II ≥ 30) [28]. Perceived body image was assessed by the question: “What do you think of your body shape?” The response options were “very skinny”, “skinny”, “normal”, “fat”, and “very fat”. The response options of subjective health status were “very healthy”, “healthy”, “moderate” “unhealthy”, and “very unhealthy”.

#### 2.3.2. Diet, Activity, and Weight Control

Behaviors related to breakfast were assessed with the question: “On how many days did you have breakfast in the last 7 days?” The response options ranged from “0 days” (1) to “7 days” (8). Another question regarding diet was as follows: “How often did you eat fast food, such as pizza, hamburger, or fried chicken, in the last 7 days?” The response options ranged from “did not eat” (1) to “3 or more times” (7). The question regarding physical activity was as follows: “In the last 7 days, on how many days did you engage in physical activity causing an increased heart rate or shortness of breath for a total of 60 min or more?” The response options were “none” (1) to “7 days” (8). A further question related to physical activity was as follows: “In the last 7 days, on how many days did you do at least 20 min of vigorous physical activity that made you short of breath or sweat?” The response options were “none” (1) to “5 days or more” (6). Attempts at weight control were assessed through the question: “In the last 30 days, have you tried to control your weight?” The response options were “no effort”, “tried to lose weight”, “tried to gain weight”, and “tried to maintain weight”.

#### 2.3.3. Drinking Behavior

Drinking behavior was measured by the question: “Have you ever drunk?” The response options were “yes” and “no”. The first drinking episode was determined with the question: “When was the first time you drank more than one glass of an alcoholic beverage?” The original response options ranged from “before entering elementary school” (1) to “third year of high school” (13). In this study, this variable was recorded as 0–12 to match the commonly used actual grade level. The number of days on which respondents drunk was determined with the question: “In the last 30 days, on how many days did you drink more than one glass of alcoholic beverage?” The response options ranged from “none” (1) to “daily” (7). The amount of alcohol consumed was determined by the question: “How much alcohol did you consume in the last 30 days?” The response options ranged from “1–2 glasses of Soju, a traditional liquor in Korea (or 1 bottle of beer or less)” (1) to “more than 2 bottles of Soju (or 8 bottles of beer or 12 glasses of liquor)” (5). The question related to blackouts was as follows: “In the last 30 days, on how many days did you drink enough to lose consciousness or experience memory loss?” The response options ranged from “none” (1) to “5 days or more” (4).

#### 2.3.4. Smoking Behavior

Smoking experience was examined with the question: “Have you ever smoked?” The response options were “yes” and “no”. The first smoking episode was determined with the question: “When did you first smoke?” The original response options ranged from “before entering elementary school” (1) to “third year of high school” (13). In this study, this variable was recorded as 0–12 to match the commonly used actual grade level. The number of days on which respondents smoked was determined with the question: “On how many of the last 30 days did you smoke even one cigarette?” The response options ranged from “none” (1) to “daily” (7). The extent of smoking was examined with the question: “In the last 30 days, on average, how many cigarettes did you smoke per day?” The response options ranged from “less than 1 cigarette/day” (1) to “more than 20 cigarettes/day” (6).

#### 2.3.5. Mental Health

Sleep satisfaction was assessed with the question: “In the last 7 days, do you think you slept enough to recover from fatigue?” The original response options ranged from “enough” (1) to “nowhere near enough” (5). Perceived stress was assessed by the question: “What level of stress do you usually feel?” The original response options ranged from “very high” (1) to “none at all” (5). In this study, sleep satisfaction and perceived stress were recorded in reverse, so higher scores indicate higher sleep satisfaction or higher stress. Depressed mood was assessed by the question: “In the last 12 months, have you ever felt so sad or hopeless that you stopped your daily activities for 2 full weeks?”, with “yes” and “no” as the response options. Suicidal ideation was characterized by a series of questions about suicidal thoughts, plans, and attempts in the last 12 months, with “yes” and “no” as the response options.

### 2.4. Data Analyses

The extracted data were analyzed using IBM SPSS Statistics software (version 24.0; IBM Corp., Armonk, NY, USA). All estimates were adjusted according to a complex sampling design. Descriptive statistics were generated including mean ± standard error (SE), range, and weighted percentages. The Rao–Scott χ2 test and Student’s *t*-test were used to identify differences in general characteristics by gender. Linear regression analysis and logistic regression were performed to identify differences in lifestyle factors and mental health according to gender by adjusting for covariates, and effect size was calculated for each test. Based on the results of bivariate analyses, general characteristics that showed a significant relation (*p* < 0.05) with the gender variable were selected as covariates (economic status, academic achievement, nutrition/eating habits education in school, drinking education in school, BMI, perceived body image, and perceived health status, Table 1).

## 3. Results

### 3.1. General Characteristics

The gender distributions of the general participant characteristics are presented in Table 1. The differences between boys and girls in general characteristics were analyzed. General characteristics except age and living with family showed differences by gender. Regarding BMI and perceived body image, more girls were underweight (boys—8.7%, girls—14.9%) and normal (boys—50.0%, girls—58.6%), and more boys were overweight (At risk: boys—15.7%, girls—13.8%; Obese I: boys—20.1%, girls—11.6%; Obese II: boys—5.5%, girls—1.1%) (Rao–Scott χ^2^ = 228.17, *p* < 0.001). However, more girls perceived themselves as normal (boys—32.2%, girls—37.0%), fat (boys—32.0%, girls—38.8%), and very fat (boys—6.5%, girls—7.6%), and more boys perceived themselves as skinny (boys—23.8%, girls—15.0%) and very skinny (boys—5.5%, girls—1.6%) (Rao–Scott χ^2^ = 224.69, *p* < 0.001).

### 3.2. Differences in Lifestyle Behaviors According to Gender

The differences in lifestyle behaviors according to gender are presented in Table 2. The gender differences in diet, physical activity, and weight control were as follows. The weighted mean ± SE of eating breakfast and eating fast food in the last week was higher among boys than girls (Wald F = 5.71, *p =* 0.017, Cohen’s *d* = 0.05 and Wald F = 33.79, *p <* 0.001, Cohen’s *d* = 0.13, respectively). The weighted mean ± SE of physical activity and vigorous physical activity in the last week was also higher among boys than girls (Wald F = 351.17, *p <* 0.001, Cohen’s *d* = 0.41, and Wald F = 353.77, *p <* 0.001; Cohen’s *d* = 0.41, respectively). Regarding weight control attempts, “no effort” (boys—55.2%, girls—52.7%) and “tried to gain weight” (boys—10.8%, girls—1.1%) were more common in boys. On the other hand, “tried to lose weight” (boys—24.6%, girls—35.3%) and “tried to maintain weight” (boys—9.4%, girls—10.9%) were more common in girls (Wald χ^2^ = 283.79, *p* < 0.001, Cohen’s *d* = 0.37).

The rate of drinking experience was higher in boys (65.2%) than girls (57.7%) (Wald χ^2^ = 15.48, *p* < 0.001, Cohen’s *d* = 0.09). Among respondents with drinking experience, the first drinking episode in boys was earlier than that in girls (Wald F = 139.24, *p <* 0.001, Cohen’s *d* = 0.26). The weighted mean ± SE of drinking days, amount drunk, and blackout experience was higher in boys (Wald F = 32.66, *p <* 0.001, Cohen’s *d* = 0.12; Wald F = 36.84, *p <* 0.001, Cohen’s *d* = 0.13; and Wald F = 5.93, *p =* 0.015, Cohen’s *d* = 0.05, respectively).

There were gender differences in smoking behavior. Smoking experience was more common in boys (31.9%) than girls (10.5%) (Wald χ^2^ = 324.67, *p* < 0.001, Cohen’s *d* = 0.40). Among respondents with smoking experience, the first smoking episode in boys was earlier than that in girls (Wald F = 45.80, *p <* 0.001, Cohen’s *d* = 0.15). The weighted mean ± SE of smoking days and amount smoked was higher in boys (Wald F = 45.93, *p <* 0.001, Cohen’s *d* = 0.15, and Wald F = 49.87, *p <* 0.001, Cohen’s *d* = 0.15, respectively).

### 3.3. Differences in Mental Health According to Gender

The weighted mean ± SE of sleep satisfaction was lower in girls (Wald F = 47.10, *p <* 0.001, Cohen’s *d* = 0.15), and perceived stress was higher in girls (Wald F = 188.71, *p <* 0.001, Cohen’s *d* = 0.30). The rate of feeling sad or hopeless enough to stop daily activities for 2 weeks in the last year was higher among girls (36.5%) than boys (25.3%) (Wald χ^2^ = 67.46, *p* < 0.001, Cohen’s *d* = 0.18). The proportion of respondents with suicidal ideation (boys—10.5%, girls—16.1%) and suicidal planning (boys—3.5%, girls—4.3%) in the last year was also higher in girls than boys (Wald χ^2^ = 24.60, *p* < 0.001, Cohen’s *d* = 0.11, and Wald χ^2^ = 8.23, *p* = 0.004, Cohen’s *d* = 0.06, respectively). There was no significant difference in suicide attempts between boys and girls (Table 3).

## 4. Discussion

The present study explored gender differences in lifestyle and mental health among senior high school students. As a result of reviewing grades 7–12 data in the 2018 KYRBS [27] report, grade 12 students had the highest scores for most negative lifestyle and mental health variables (breakfast missing rate, fast food consumption rate, obesity rate, lifetime/current drinking rate, risk drinking rate, drunken experience rate, lifetime/current smoking rate, stress perception rate, and depression experience rate). On the other hand, in the positive variables (average sleep time per week, subjective sleep satisfaction rate, steady/vigorous physical activity rate), senior high school students had the lowest value. In a previous study exploring the quality of sleep of senior high school students, most of them (74.8%) answered that they had no sleep problems before they started preparing for college entrance exams [12]. These findings suggest the possibility that college entrance exam preparation may become a threatening factor to the lifestyle and mental health of senior high school students.

In this study, girls were more likely to be underweight, and less likely to be overweight, than boys, but they tended to perceive themselves as being fatter than they actually were. A population-based study of Japanese adolescents also found that boys tended to underestimate their own weight, while girls tended to overestimate it [29]. In this study, the girls also perceived their overall health more negatively. This was consistent with the findings of a study exploring the self-assessed health of Swedish adolescents, where the proportion of adolescents reporting good health was lower for girls than for boys [30]. In a study of Spanish high school students, higher body dissatisfaction and more negative self-identity were found in girls, which were related to low self-esteem and low intrinsic motivation [31]. Adolescence is a critical period with respect to development of the body and self-identity, and there is a need for more research on why girls have more negative perceptions about their body and health.

Lifestyle behaviors related to diet, exercise, and weight control in this study varied by gender. The boys ate breakfast more frequently and engaged in more physical activity than the girls. The boys ate breakfast and fast food more frequently, were more physically active, and tried to gain weight more, along with having higher rates of obesity. Previous studies of Kuwaiti and American adolescents also reported that boys were more physically active, ate more food, and attempted to gain weight more [32,33]. Meanwhile, the girls ate less fast food, but ate breakfast less frequently, were less physically active, and tried to lose weight more. Some previous studies also reported that girls were less engaged in physical activity and consumed more vegetables instead of high-calorie foods; these factors were associated with lower levels of body satisfaction [24]. Girls’ nutrition and physical activity are influenced by norms and ideals related to gender roles and body centrality [34]. Girls under significant social pressure to have a beautiful body may be more focused on creating an ideal body than following a healthy lifestyle. A study of Turkish adolescents indicated gender differences not only in body perception but also in strategies for coping with body-related stress [35]. Negative body image or body image distortion were found to be associated with unhealthy eating habits, low self-esteem, sadness, depression, and suicidal ideation in adolescents [36,37]. Therefore, considering gender differences, interventions to improve body image and maintain an appropriate weight will be more effective in improving the physical and mental health of senior high school students.

In this study, boys showed higher rates of drinking and smoking. Compared to girls, boys were more likely to drink alcohol, had an earlier first drinking experience, drank on more days, consumed more alcohol per drink, and experienced more blackouts. The same patterns were seen in their smoking behavior. A large European study found gender differences in late adolescence, where boys drank more frequently and got drunk more often than girls [38]. According to a 2012–2017 literature review of gender differences in e-cigarette use among US adolescents, boys appear to use them more frequently, although girls may be more vulnerable to e-cigarette advertising, given that this was shown for cigarettes [39]. Similar results were reported in a large study of adolescents in China [40]. Previous studies on drinking and smoking among adolescents according to gender indicated that boys are highly influenced by social factors and peers, while drinking and smoking are highly associated with depression in girls [38,40,41,42]. Considering these gender differences related to drinking and smoking among senior high school students will help reduce their health risk behaviors, especially among boys.

In this study, the girls had a poorer sleep quality, higher stress levels, a more depressed mood, and more suicidal ideations and plans. In a study that investigated gender differences in the mental health of a representative sample of Belgian adolescents, girls reported significantly higher levels of psychological distress, anxiety, and depression [43]. In general, women are more prone to depression than men, regardless of the culture [44]. In addition, women are more likely to experience physical (somatic) symptoms of depression, such as disturbances in appetite and sleep, while men are much more likely to commit suicide [45]. A systematic review and meta-analysis of longitudinal studies of gender differences in suicidal behavior among adolescents showed that women had higher rates of suicide attempts, but men were at higher risk of successful suicide [46]. A study based on data from the Chinese Youth Risk Behavior Survey showed that girls were more prone to suicidal thoughts than boys and reported higher levels of academic pressure, smoking, binge drinking, and fighting [9]. According to two meta-analyses of a nationally representative sample, gender differences in depression rates appear as early as 12 years of age and peak in adolescence [47]. Higher sensitivity to stress, loss of interpersonal or emotional closeness, and victimization of violence have been reported as gender-specific risk factors for depression and suicidal behavior that are higher in girls [46,48]. In a study of gender differences in predictors of depression among multicultural adolescents in South Korea, depression in girls was predicted by low self-esteem, level of social withdrawal, low school life satisfaction, prevalence of neglectful parenting style, and perceived disinterest of parents [49]. As girls are more likely to be diagnosed with mental health problems, beginning in early adolescence, early interventions promoting mental health in girls are required. In future, it will be necessary to investigate the factors underlying gender differences in mental health and subjective experiences.

The data used in this study were generated by a cross-sectional survey method, and thus the understanding of causality is limited. As a secondary analysis of national public data, this study has measurement limitations due to the fact that the verified mental health questionnaire was not used and psychometric information was not provided from the data source. Additionally, this study used online self-reported data, which may have been affected by response bias. In the future, given reports of the lifestyle and health impacts of the COVID-19 pandemic, and gender differences therein [1,13,50,51], it will be necessary to longitudinally explore the effects of global pandemics and associated control measures on the lives of young people.

## 5. Conclusions

According to our secondary analysis of data on the health behaviors of senior high school students in South Korea, boys were more likely to engage in risky health behaviors, whereas girls were more likely to suffer from poor mental health and exhibit certain negative lifestyle behaviors. To maintain a healthy lifestyle and good mental health among adolescents, a positive body image is necessary, especially for girls. Preventive measures and interventions for health risk behaviors in adolescents should take into account factors related to drinking and smoking by gender, with a particular focus on boys. Our findings suggest that education and health institutions should take account of the differences between boys and girls so that their needs can be met. Gender-specific approaches are required to promote healthy lifestyles and good health status among senior high school students.

## Figures and Tables

**Table 1:** General characteristics of the participants (*n* = 8476).

Variables	Boys (*n* = 4131, 51.0% *)	Girls (*n* = 4345, 49.0% *)	Rao–Scott χ^2^ or t (*p*)
	*n* (% *) or M * ± SE		
Age (y) ^†^	17.45 ± 0.01 (Range: 16–18)	17.44 ± 0.01 (Range: 16–18)	0.11 (0.914)
Living with family			
No	346(7.2)	400 (7.7)	0.65 (0.630)
Yes	3785 (92.8)	3945 (92.3)	
Economic status			
High	433 (10.6)	265 (6.3)	53.51 (<0.001)
Medium-high	1104 (27.0)	1232 (28.7)	
Medium	1904 (46.1)	2144 (49.3)	
Medium-low	548 (13.0)	571 (12.7)	
Low	142 (3.3)	133 (3.0)	
Academic achievement ^††^			
High	482 (11.4)	346 (8.0)	59.89 (<0.001)
Medium-high	890 (21.2)	1006 (23.1)	
Medium	1235 (30.3)	1448 (33.6)	
Medium-low	976 (23.6)	1098 (25.2)	
Low	548 (13.5)	447 (10.1)	
Education in school			
Nutrition/eating habits ^††^			
No	2707 (66.0)	3150 (73.2)	51.88 (<0.001)
Yes	1424 (34.0)	1195 (26.8)	
Drinking ^††^			
No	2840 (69.8)	3212 (75.0)	28.48 (<0.001)
Yes	1291 (30.2)	1133 (25.0)	
Smoking ^††^			
No	1694 (42.2)	1718 (40.5)	2.63 (0.334)
Yes	2437 (57.8)	2627 (59.5)	
Body mass index (BMI) ^†^			
Underweight (<18.5)	352 (8.7)	621 (14.9)	228.17 (<0.001)
Normal (≥18.5, <23.0)	1972 (50.0)	2481 (58.6)	
Overweight	1653 (41.3)	1116 (26.5)	
At risk (≥23.0, <25.0)	632 (15.7)	583 (13.8)	
Obese I (≥25.0, <30.0)	796 (20.1)	487 (11.6)	
Obese II (≥30)	225 (5.5)	46 (1.1)	
Perceived body image			
Very skinny	226 (5.5)	71 (1.6)	224.69 (<0.001)
Skinny	973 (23.8)	650 (15.0)	
Normal	1341 (32.2)	1614 (37.0)	
Fat	1305 (32.0)	1670 (38.8)	
Very fat	286 (6.5)	340 (7.6)	
Perceived health status			
Very healthy	1258 (29.9)	643 (14.8)	325.48 (<0.001)
Healthy	1670 (39.8)	1888 (43.5)	
Moderate	883 (22.2)	1233 (28.2)	
Unhealthy	288 (7.3)	549 (12.8)	
Very unhealthy	32 (0.8)	32 (0.7)	

M = mean; SE = standard error; R = range; * weighted; ^†^ excluded missing values; ^††^ in the last year.

**Table 2:** Differences in lifestyle behaviors according to gender (*n* = 8476).

Variables	Boys (*n* = 4131, 51.0% *)	Girls (*n* = 4345, 49.0% *)	Wald F ^#^ or Wald χ^2^^#^ (*p*)	Cohen’s *d*
	*n* (% *) or M * ± SE			
Eating breakfast (days) ^†^	5.04 ± 0.10	4.86 ± 0.10	5.71 (0.017)	0.05
Eating fast food (times) ^†^	2.32 ± 0.04	2.19 ± 0.04	33.79 (<0.001)	0.13
Physical activity (days) ^†^	3.16 ± 0.07	2.06 ± 0.07	351.17 (<0.001)	0.41
Vigorous physical activity (days) ^†^	3.18 ± 0.06	2.14 ± 0.06	353.77 (<0.001)	0.41
Weight control attempts ^‡^				
No effort	2281 (55.2)	2284 (52.7)	283.79 (<0.001)	0.37
Tried to lose weight	1001 (24.6)	1534 (35.3)		
Tried to gain weight	443 (10.8)	51 (1.1)		
Tried to maintain weight	406 (9.4)	476 (10.9)		
Drinking experience				
No	1444 (34.8)	1832 (42.3)	15.48 (<0.001)	0.09
Yes	2687 (65.2)	2513 (57.7)		
First drinking episode ^††^	8.17 ± 0.14	8.99 ± 0.14	139.24 (<0.001)	0.26
Drinking days ^‡,††^	2.00 ± 0.07	1.78 ± 0.78	32.66 (<0.001)	0.12
Amount drunk (glasses) ^§^	3.25 ± 0.09	2.80 ± 0.11	36.84 (<0.001)	0.13
Blackout experience ^§^	1.38 ± 0.06	1.32 ± 0.60	5.93 (0.015)	0.05
Smoking experience				
No	2816 (68.1)	3885 (89.5)	324.67 (<0.001)	0.40
Yes	1315 (31.9)	460 (10.5)		
First smoking episode ^||^	7.09 ± 0.17	8.11 ± 0.22	45.80 (<0.001)	0.15
Smoking days ^‡,||^	3.56 ± 0.18	2.69 ± 0.20	45.93 (<0.001)	0.15
Amount smoked (no. cigarettes) ^¶^	3.73 ± 0.15	2.90 ± 0.19	49.87 (<0.001)	0.15

* Weighted; ^†^ in the last week; ^‡^ in the last month; ^††^ among respondents with drinking experience; ^§^ among respondents with at least 1 drinking day in the last month; ^||^ among respondents with smoking experience; ^¶^ among respondents with at least 1 smoking day in the last month; ^#^ the results were adjusted for covariates (economic status, academic achievement, nutrition/eating habits education in school, drinking education in school, body mass index, perceived body image, and perceived health status).

**Table 3:** Differences in mental health according to gender (*n* = 8476).

Variables	Boys (*n* = 4131, 51.0% *)	Girls (*n* = 4345, 49.0% *)	Wald F ^#^ or Wald χ^2^^#^ (*p*)	Cohen’s *d*
	*n* (% *) or M * ± SE			
Sleep satisfaction ^†,††^	2.41 ± 0.04	2.21 ± 0.04	47.10 (<0.001)	0.15
Perceived stress	3.62 ± 0.02	3.92 ± 0.03	188.71 (<0.001)	0.30
Depressed mood ^§^				
No	3111 (74.7)	2764 (63.5)	67.46 (<0.001)	0.18
Yes	1020 (25.3)	1581 (36.5)		
Suicidal characteristics				
Suicidal ideation ^§^				
No	3711 (89.5)	3646 (83.9)	24.60 (<0.001)	0.11
Yes	420 (10.5)	699 (16.1)		
Suicidal planning ^§^				
No	3988 (96.5)	4155 (95.7)	8.23 (0.004)	0.06
Yes	143 (3.5)	190 (4.3)		
Suicide attempt ^§^				
No	4041 (97.7)	4226 (97.4)	3.58 (0.058)	0.04
Yes	90 (2.3)	119 (2.6)		

* Weighted; ^†^ excluding missing values; ^††^ in the last week; ^§^ in the last year; ^#^ the results were adjusted for covariates (economic status, academic achievement, nutrition/eating habits education in school, drinking education in school, body mass index, perceived body image, and perceived health status).
